# Cell surface Thomsen-Friedenreich proteome profiling of metastatic prostate cancer cells reveals potential link with cancer stem cell-like phenotype

**DOI:** 10.18632/oncotarget.21985

**Published:** 2017-10-20

**Authors:** Feng Li, Olga V. Glinskii, Brian P. Mooney, Kate Rittenhouse-Olson, Kenneth J. Pienta, Vladislav V. Glinsky

**Affiliations:** ^1^ Research Service, Harry S. Truman Memorial Veterans Hospital, Columbia, MO, USA; ^2^ Department of Pathology and Anatomical Sciences, University of Missouri, School of Medicine, Columbia, MO, USA; ^3^ Department of Medical Pharmacology and Physiology, University of Missouri, School of Medicine, Columbia, MO, USA; ^4^ Department of Biochemistry and Charles W. Gehrke Proteomics Center, University of Missouri-Columbia, Columbia, MO, USA; ^5^ Department of Biotechnical and Clinical Laboratory Sciences and the Department of Microbiology, University at Buffalo, Buffalo, NY, USA; ^6^ For-Robin, Inc, Buffalo, NY, USA; ^7^ Department of Urology, The James Buchanan Brady Urological Institute, Departments of Oncology and Pharmacology and Molecular Sciences, The Johns Hopkins School of Medicine, Baltimore, MD, USA

**Keywords:** Thomsen-Friedenreich antigen, proteomics, tumor metastasis, cancer stem cells, prostate cancer

## Abstract

The tumor-associated Thomsen-Friedenreich glycoantigen (TF-Ag) plays an important role in hematogenous metastasis of multiple cancers. The LTQ Orbitrap LC-MS/MS mass spectrometry analysis of cell surface TF-Ag proteome of metastatic prostate cancer cells reveals that several cell surface glycoproteins expressing this carbohydrate antigen in prostate cancer (CD44, α2 integrin, β1 integrin, CD49f, CD133, CD59, EphA2, CD138, transferrin receptor, profilin) are either known as stem cell markers or control important cancer stem-like cell functions. This outcome points to a potential link between TF-Ag expression and prostate cancer stem-like phenotype. Indeed, selecting prostate cancer cells for TF-Ag expression resulted in the enrichment of cells with stem-like properties such as enhanced clonogenic survival and growth, prostasphere formation under non-differentiating and differentiating conditions, and elevated expression of stem cell markers such as CD44 and CD133. Further, the analysis of the recent literature demonstrates that TF-Ag is a common denominator for multiple prostate cancer stem-like cell populations identified to date and otherwise characterized by distinct molecular signatures. The current paradigm suggests that dissemination of tumor cells with stem-like properties to bone marrow that occurred before surgery and/or radiation therapy is largely responsible for disease recurrence years after radical treatment causing a massive clinical problem in prostate cancer. Thus, developing means for destroying disseminated prostate cancer stem-like cells is an important goal of modern cancer research. The results presented in this study suggest that multiple subpopulation of putative prostate cancer stem-like cells characterized by distinct molecular signatures can be attacked using a single target commonly expressed on these cells, the TF-Ag.

## INTRODUCTION

Thomsen-Friedenreich antigen (TF antigen, TF-Ag, T antigen, CD176) is a core 1 glycan structure Galβ1–3GalNAcα1- O-linked to serine or threonine, which is commonly present in a cryptic form on many normal cell types, but is unmasked and reactive on the vast majority (about 90%) of all human cancers including colon, breast, bladder, prostate, liver, ovary and stomach [1]. Previous works from this and other groups demonstrated the role for TF-Ag in metastatic spread of breast [2, 3], prostate [2, 4, 5], colon [6], and pancreatic cancer [7]. Specifically, TF-Ag has been implicated in mediating metastatic cell adhesive interactions with vascular endothelium [2, 6, 8, 9] as well as homotypic tumor cell aggregation [10], thus controlling important rate-limiting steps in cancer metastasis [11]. Two recent groundbreaking studies highlight the critical role played in estrogen receptor-negative breast cancer metastasis by the enzyme ST6GalNAc2 controlling the expression of TF-antigen [12], and by C2GnT2 and ST6GalNAc4 glycosyltransferase activity in lung adenocarcinoma controlling metastatic cell attachment to galectin-3-presenting cells in the metastatic niche via regulation of TF antigen expression [13].

To date, in various types of cancer TF antigen has been shown to be expressed on several glycoproteins. Specifically, the mucin MUC1 has been shown to express TF-Ag in breast [14] and colon [6] while MUC4 in pancreatic [7] cancer; CD44 has been identified as a major TF-Ag carrier in colon cancer [15]; in cancers of lung, breast, and liver CD133 and CD44 express TF-Ag [16]; and CD34 was shown to express TF-Ag on malignant hematopoietic cells [17]. In prostate cancer, however, the TF proteome has not been investigated systematically until now. Thus, in this study we have performed the analysis of cell surface prostate cancer TF proteome. Using the affinity purification of biotinylated cell surface proteins followed by a pull-down with TF-Ag specific peanut agglutinin (PNA) and a shotgun mass spectrometry analysis as well as immunoprecipitation (IP) of individual glycoproteins, and Western blot verification of TF-Ag expression, we unambiguously identified 18 cell surface glycoproteins as TF-Ag carriers expressed on PC-3 and DU-145 metastatic human prostate carcinoma cells. Remarkably, several of these TF-Ag expressing glycoproteins namely CD44, α2β1 integrin, CD133, CD49f (α6 integrin), and ephrin type-A receptor 2 (EphA2) either serve as most commonly used cell surface prostate cancer stem cell markers (CD44, α2β1 integrin, CD133, CD49f) or control stem cell like functions including clonogenic potential, self-renewal, prostasphere formation, tumor onset, and dissemination of prostate carcinoma to the skeleton (EphA2) [18–27].

Indeed, selecting TF-Ag expressing PC-3 prostate cancer cells yielded a subpopulation of cells with enhanced stem cell properties such as clonogenic survival, clonogenic growth, prostasphere formation under both non-differentiating and differentiating conditions, and enhanced expression of stem cell markers compared with their TF-Ag negative counterparts. Taken together, these results strongly suggest that TF antigen expression in prostate cancer cells is associated with the stem cell like phenotype. It appears that TF antigen could be a common denominator for multiple populations of prostate cancer stem like cells identified to date and otherwise characterized by distinct cell surface signatures [18]. Consequently, TF antigen specific therapeutics such as anti-TF antibodies [28] or TF-Ag binding peptides [29] could potentially provide means for targeting simultaneously multiple prostate cancer stem cell subtypes.

## RESULTS

### Cell surface metastatic prostate cancer cell TF proteome

### Two-step affinity purification and LTQ orbitrap LC-MS/MS analysis of cell surface TF-ag expressing glycoproteins

In the present work, we employed a method combining biotinylation of cell surface proteins followed by a streptavidin agarose pull-down, lectin affinity chromatography, and LC-MS/MS mass spectrometry analysis to identify cell surface TF-Ag glycoprotein carriers expressed on PC-3 and DU-145 human prostate carcinoma cell lines.

The cell surface proteins were first labeled by Sulfo-NHS-SS-Biotin, followed by the affinity purification using streptavidin-coated agarose beads. Next, upon reduction with DTT, disulfide bonds were broken to release biotinylated proteins from the beads [30], which were further purified using affinity chromatography with peanut agglutinin (PNA-lectin), having high specific capability to bind peptides with TF-Ag glycan structure, to capture the TF-Ag expressing glycoproteins.

**Figure 1:**
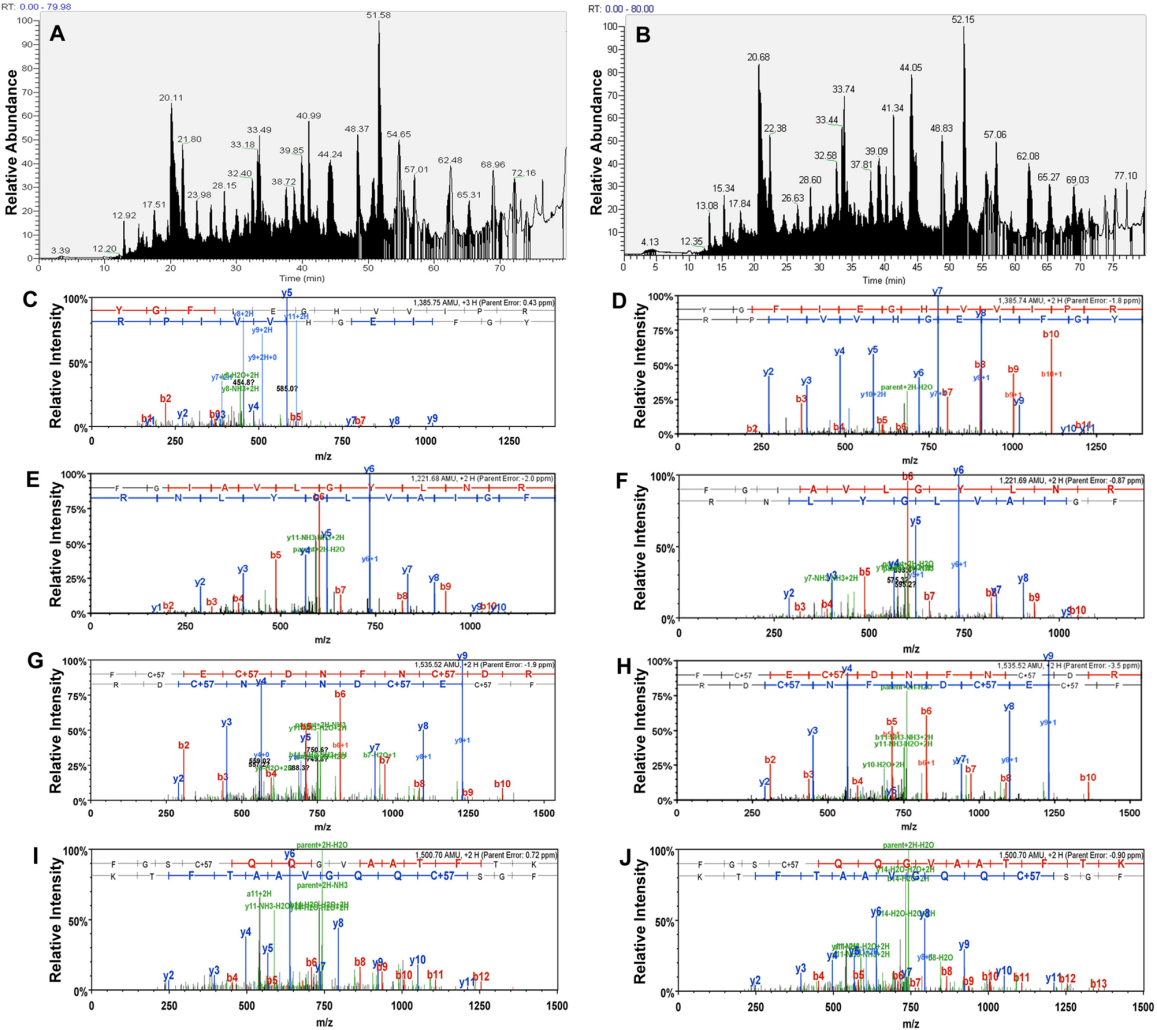
Shotgun proteomics and LC MS/MS analysis (**A**) and (**B**), Representative base peak chromatograms of PNA-lectin captured fraction from DU-145 (A) and PC-3 (B) cells. (**C**) and (**D)**, Examples of MS/MS spectra of the same peptide from CD44 identified in DU-145 (C) and PC-3 (D) cells. The sequence of the peptide was identified as YGFIEGHVVIPR. (**E**) and (**F**), MS/MS spectra of the same peptide from the integrin alpha-2 identified in DU-145 (E) and PC-3 (F) cells. The sequence of the peptide was identified as FGIAVLGYLNR. (**G**) and (**H)**, MS/MS spectra of the same peptide from the integrin beta-1 identified in DU-145 (G) and PC-3 (H) cells. The sequence of the peptide was identified as FCECDNFNCDR. (**I)** and (**J)**, MS/MS spectra of the same peptide from the integrin alpha-6 identified in PC-3 (I) and DU-145 (J) cells. The sequence of the peptide was identified as FGSCQQGVAATFTK.

The enriched TF-Ag carrying glycoproteins were digested and analyzed by LC-MS/MS using LTQ Orbitrap mass spectrometry. Figure 1 shows representative base peak chromatograms of the PNA lectin captured fractions from PC-3 and DU-145 cell surface proteins, as well as examples of MS/MS spectra of the same peptides from CD44, α2 integrin, β1 integrin, and α6 integrin identified in samples from PC-3 and DU-145 cells.

In three independent experiments, the LTQ Orbitrap analysis resulted in the identification of 45, 152, and 97 proteins from biotinylated PNA-lectin tandem affinity purification fractions from DU-145 cells. Similarly, 123, 39, 102, and 102 proteins were identified in four independent experiments with PC-3 cells. However, due to the limitations of immunoprecipitation technique and high sensitivity of the LTQ Orbitrap mass spectrometry, not all of the proteins identified in these experiments are cell surface TF-Ag carriers. A significant number of them could be either non-specifically absorbed high abundance intracellular proteins or co-precipitated proteins interacting with the TF-Ag expressing glycopeptides. Thus, our next task was to screen out such proteins and select only cell surface glycoproteins. To achieve this, a consensus transmembrane prediction strategy combining the prediction results from several different transmembrane prediction software tools was used.

### Consensus transmembrane prediction analysis

A consensus prediction strategy employing several transmembrane prediction tools has been proven to improve overall accuracy compared to any of the individual methods. [31]. Here, three transmembrane prediction software tools were used, including ones based upon simple hydrophobicity analysis (TopPred), thermodynamic and biological principles (MPEx), and hidden Markov models (TMHMM) [32–34]. First, one dataset generated from DU-145 cell line experiment number two including 152 identified proteins was used to evaluate the accuracy of these three transmembrane prediction software tools. All software tools were set up to the default settings. Compared to MPEx and TopPred, TMHMM predicted 18 transmembrane proteins with at least one transmembrane segment, which was the least number of predicted transmembrane proteins among the three prediction results. MPEx and TopPred had significantly more predicted transmembrane proteins with at least one transmembrane segment. However, the analysis yielded almost the same number of transmembrane proteins when the threshold was set to two transmembrane segments in MPEx and three transmembrane segments in TopPred. TMHMM had the highest conservation and accuracy, which was coincident with the literature. Thus, for the final analysis, the threshold of transmembrane proteins was set to one transmembrane segment in TMHMM, two transmembrane segments in MPEx and three transmembrane segments in TopPred in all datasets.

**Figure 2:**
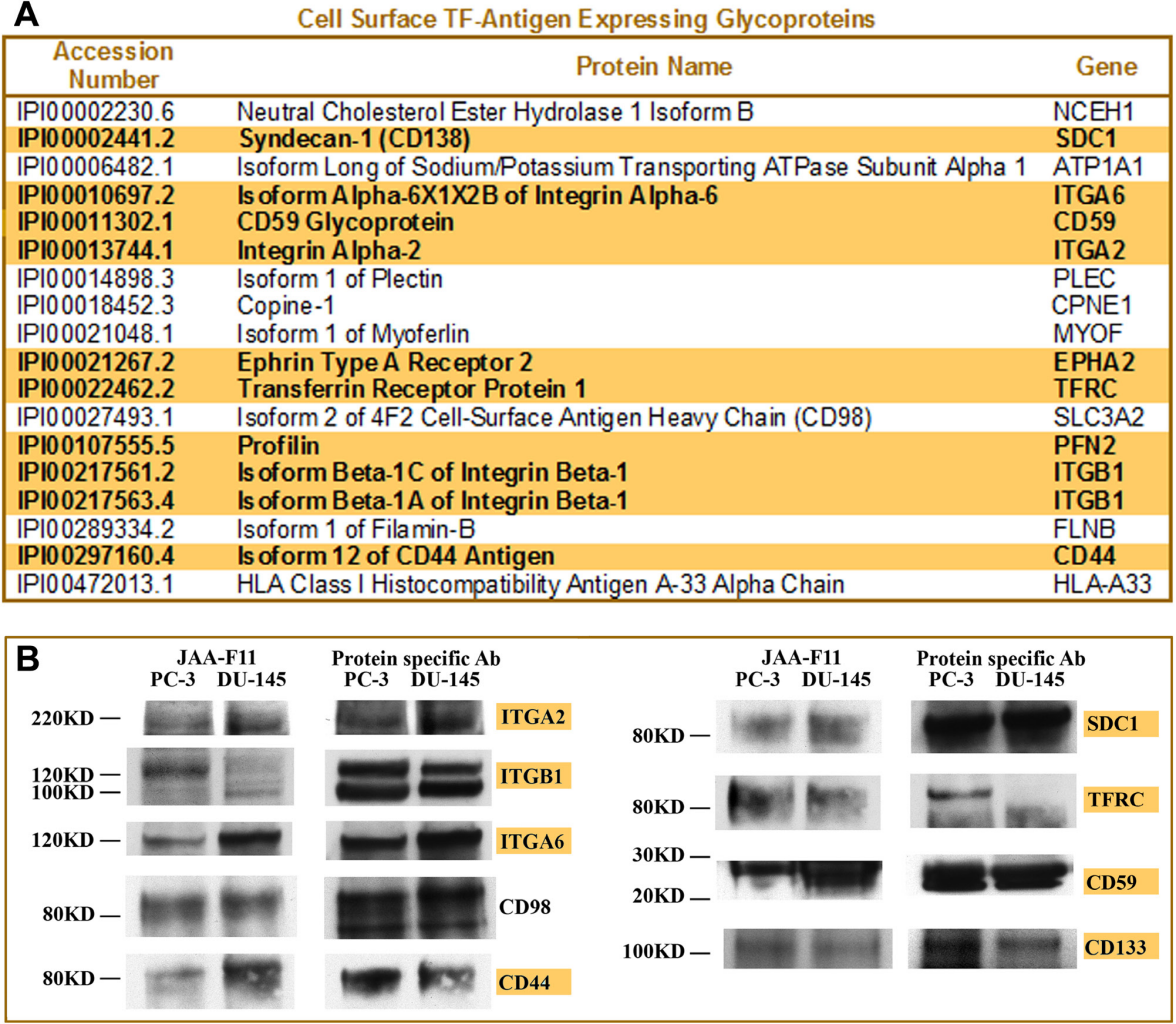
Identification of cell surface glycoproteins expressing TF-Ag in prostate cancer (**A**) List of cell surface glycoproteins expressing TF-Ag identified by LTQ Orbitrap LC MS/MS analysis of PNA-labeled fractions from DU-145 and PC-3 cells. Glycoproteins highlighted in bold are known as prostate cancer stem cell markers or controlling important cancer stem-like cell functions. (**B**) Confirmation of TF-Ag expression by IP and Western analysis. Individual glycoproteins from PC-3 and DU-145 cells were immunoprecipitated, resolved by PAG electrophoresis, transferred to the nitrocellulose membrane, and probed with anti-TF-Ag mAb JAA-F11 for TF-Ag expression. To verify a proper position of TF-Ag-reactive bands, membranes were stripped and reprobed with protein-specific Abs used for IP. Names of the proteins known as prostate cancer stem cell markers or controlling important cancer stem-like cell functions are highlighted in yellow.

Although TMHMM has the highest accuracy to predict transmembrane proteins among these three prediction software tools, the consensus strategy can provide a better result than any single prediction method alone. For example, isoform 1 of plectin was predicted as a non-transmembrane segment containing by TMHMM, but as having two transmembrane segments by MPEx and three transmembrane segments by TopPred. Thus, based on the consensus prediction strategy, plectin should be considered as protein with transmembrane segments, which is consistent with the published literature [35]. All positive transmembrane prediction results were manually validated and imported into a desktop Microsoft Access database. The proteins that appeared at least twice in all datasets were considered TF-antigen expressing cell surface proteins and listed in Figure 2A. The proteins appearing in all datasets only once were considered as possible TF-antigen expressing proteins and listed in Supplemental Table 1 as well as TF-Ag interacting and mucin-like proteins identified by the LTQ Orbitrap analysis.

One remarkable outcome of this analysis was that a significant fraction of glycoproteins identified as cell surface TF-antigen carriers in PC-3 and DU-145 cells (Figure 2A) are associated with stem cell phenotype or function, including prostate cancer stem-like cells. Specifically, CD44, α2 integrin, β1 integrin, and CD49f (α6 integrin) are the most commonly recognized prostate cancer stem cell markers, while EphA2 controls prostate cancer stem cell-like functions such as clonogenic potential, self-renewal, prostasphere formation, tumor onset, and dissemination of prostate carcinoma to the bone [18–27], and syndecan-1 (CD138) stabilizes prostate cancer tumor-initiating cells [36]. In addition, CD59 is one of the most commonly reported positive markers for adipose-derived stem cells [37] and human mesenchymal stem cells [38]; transferrin receptor is preferentially required and overexpressed by glioblastoma stem-like cells [39]; and profilin is required for germline stem cell maintenance [40] and promotes migration, invasion, and stemness of human colorectal cancer stem cells [41].

### Western blot verification of TF-Ag expression

To validate the mass spectrometry identified TF-Ag expressing cell surface proteins, eight of the identified glycoproteins (integrin α2, integrin β1, integrin α6, CD98, CD44, Syndecan-1, TFRC and CD59) were individually immunoprecipitated, resolved by SDS PAGE, and probed with anti-TF-Ag monoclonal antibody JAA-F11 [42] using Western blot techniques. In addition, yet another commonly recognized stem cell marker (including prostate cancer stem cells) CD133, which is expressed at rather low levels and could be often masked by more abundant proteins in shotgun mass spectrometry analyses, was also immunoprecipitated and analyzed for TF antigen expression by Western blot. After probing with TF-Ag-specific antibody JAA-F11, the membranes were stripped and reprobed with corresponding protein-specific antibodies to confirm the correct position of TF-Ag-positive bands. The results of these experiments confirmed that all selected glycoproteins including CD133 do indeed express TF antigen (Figure 2B).

### Selection and characterization of TF antigen positive prostate cancer cells

### TF antigen positive prostate cancer cells exhibit enhanced clonogenic survival and clonogenic growth ability compared to their TF antigen negative counterparts

The fact that the majority of prostate cancer cell surface TF-Ag-expressing glycoproteins are known as stem cell markers prompted us to ask a question whether selecting TF-Ag-positive prostate cancer cells will enrich for the cells with stem-like properties. To interrogate this question, we have used magnetic activated cell sorting (MACS) with biotinylated anti-TF-Ag antibody JAA-F11 to isolate TF-Ag-positive (TF+) and TF-Ag-negative (TF-) prostate cancer cells. Of note, in multiple isolation experiments, the average yield of TF+ cells was 3.93% ± 0.901% for PC-3 and 0.36% ± 0.21% for DU-145 cells (mean ± standard error of mean).

**Figure 3:**
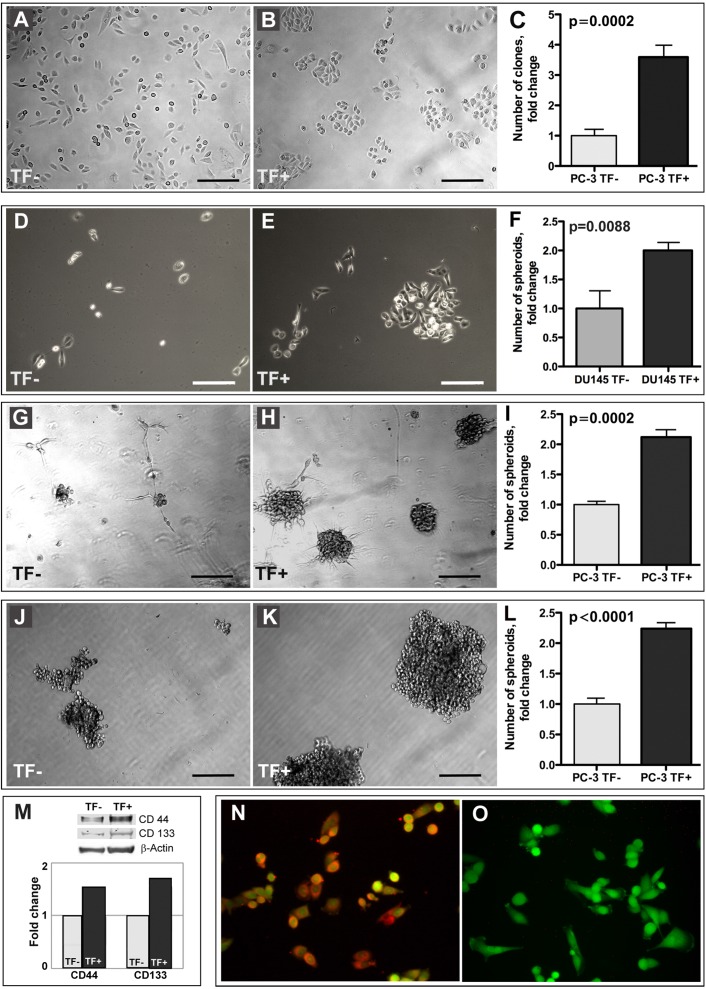
Stem-like properties of TF+ cells Unlike TF- cells (**A**), TF+ population (**B**) of PC-3 cells exhibits growth patterns characteristic of clonogenic growth even when plated at the regular densities and culturing conditions. (**C**) TF+ PC-3 cells form 3.5-fold more clones than TF- cells, when plated at the low density (200 cells per well in a 24-well plate) for clonogenic survival. Similarly, TF+ DU-145 cells exhibit enhanced ability to clonogenic survival and growth compared to TF- cells (**D)** and (**E**). TF+ DU-145 cells form 2-fold more prostaspheres than their TF- counterparts (**F**). Compared to TF- cells (**G**), TF+ population (**H**) of PC-3 cells forms > 2-fold more prostaspheres (**I**) of a much bigger size, when cultured for 2 weeks in Matrigel using serum free media. Compared to TF- cells (**J**), TF+ population (**K**) of PC-3 cells forms > 2-fold more prostaspheres (**L**) of a much bigger size, when cultured for 2 weeks in ultra-low attachment plates using complete media. (**M**) Western blot analysis of TF+ and TF-.PC-3 cells. Note higher expression of stem cell markers CD44 and CD133 by TF+ cells. (**N** and **O**) Immunofluorescent analysis of TF-Ag expression on CD44 positive subpopulation of PC-3 cells using anti-TF-Ag antibody JAA-F11. N, 100% of CD44+ cells express TF-Ag. O, Negative control omitting primary antibody. In K and L, red - TF-Ag; green - GFP. In C, F, I, L, and M, the results are normalized to TF- cells. In A, B, D, E, G, H, J and K, bar, 200 μm.

One of the fundamental unique properties attributed to stem cells, including prostate cancer stem-like cells, is self-renewal. There are several commonly accepted tools for analyzing putative stem-like cells self-renewal capacity including clonogenic assay, which is based on seeding a small number of cells and monitoring colony formation over a defined time period. Remarkably, in contrast to TF- cells, the TF+ population exhibited clonogenic growth patterns even when plated at the regular densities and culturing conditions (Figure 3A and 3B). When plated at the low density (200 cells per well in a 24-well plate) for clonogenic survival and growth, TF+ PC-3 cells formed 3.5-fold more clones than TF- cells did, (Figure 3C). Similarly, TF+ DU-145 cells exhibited stronger propensity to clonogenic survival and growth than their TF- counterparts (Figure 3D and 3E) indicating that TF-Ag selection does enrich for prostate cancer cells with enhanced clonogenicity.

### TF antigen positive prostate cancer cells form significantly more and larger prostaspheres under both non-differentiating and differentiating conditions

Another approach to analyzing self-renewal capacity of cancer stem-like cells is the sphere formation assay. The sphere formation assay is based on low adherence cultures in defined serum-free medium (non-differentiating conditions), or serum-supplemented medium (differentiating conditions) to produce multicellular spheroid aggregates called tumorspheres (prostaspheres in prostate cancer), which are enriched for cells with stem-like phenotypes.

In our experiments, under non-differentiating conditions (serum free cultures in Matrigel) TF+ PC-3 cells formed greater than 2-fold more prostaspheres of a much bigger size than TF- cells did (Figure 3G–3I). Under differentiating conditions (complete media in ultra-low attachment plates), TF+ PC-3 cells also formed greater than 2-fold more prostaspheres than TF- cells of even bigger size (Figure 3J–3L). Similarly, TF+ DU-145 cells formed 2-fold more prostaspheres than TF- cells (Figure 3F). The results of these experiments demonstrated that TF+ cells possess far superior prostasphere forming ability compared to TF- cells.

### TF antigen positive prostate cancer cells express higher levels of stem cell markers

As TF+ prostate cancer cells demonstrated much higher ability for clonogenic survival and growth, as well as the propensity to form prostaspheres under both differentiating and non-differentiating conditions compared to TF- cells, our next question was whether TF-Ag selection leads to the enrichment of cells with elevated expression of stem cell markers such as CD44 and CD133. The comparison of CD44 and CD133 expression in TF+ and TF- cells by Western blot analysis revealed that TF+ cells express significantly higher levels of both CD44 and CD133 (Figure 3M). Further, when we reversed the experiment and selected using MACS techniques CD44 positive prostate cancer cells, the immunofluorescent analysis demonstrated that practically 100% of CD44+ cells express TF-Ag (Figure 3N and 3O).

## DISCUSSION

The results of LTQ Orbitrap LC-MS/MS mass spectrometry analysis of prostate cancer cell surface TF-Ag proteome presented in this study demonstrate that many of TF-Ag expressing cell surface glycoproteins in prostate cancer cells are in fact either well known stem cell markers (CD44, α2 integrin, β1 integrin, CD49f, CD59) or the proteins controlling important cancer stem-like cell functions (EphA2, CD138, transferrin receptor, profilin). This outcome strongly suggests that there is a link between TF-Ag expression and prostate cancer stem-like phenotype. Indeed, selecting prostate cancer cells for TF-Ag expression resulted in the enrichment of cells with stem-like properties characterizing self-renewal capacity such as enhanced clonogenic survival and growth (Figure 3A–3E) and prostasphere formation under both non-differentiating and differentiating conditions (Figures 3F–3L), as well as increased expression of stem cell markers CD44 and CD133 (Figure 3M). Obviously, there are multiple mechanistic questions to be answered regarding the association between TF-Ag expression and cancer stem-like phenotype. However, there is one practical implication of this study requiring an immediate attention. The fact that various stem cell markers in prostate cancer express TF-Ag offers unparalleled opportunity for therapeutic targeting of multiple subpopulations of prostate cancer stem-like cells. Indeed, a simple analysis of the cell surface signatures discovered in recent years for putative prostate cancer stem cells by different groups (Table 1) reveals that every one of these signatures contains at least one, or two, or even three TF-Ag glycoprotein carriers.

**Table 1:** Cell surface marker signatures used recently to characterize putative prostate cancer CSC populations (markers in bold express TF-Ag in prostate cancer cells)

Signature	CSC Source/Model	Reference
CD44+/CD24^−^	Cell lines and xenografts	Hurt EM et al. British Journal of Cancer 2008, 98:756–765 [46]
CD44^+^/α2β1^hi^/CD133^+^	Primary tumors	Collins AT et al. Cancer Res 2005; 65:10946–10951 [27]
CD44^+^/α2β1^+^	DU145, LAPC4, LAPC9	Patrawala L et al. Cancer Res 2007; 67:6796–6805 [47]
CD133^hi/^/CD44^hi^/OCT4^hi^	NHPrE1	Jiang M et al. Stem Cells 2010; 28:344–56 [48]
CD49f^hi^/Trop2^hi^	Primary prostate tissue	Goldstein AS et al. Science 2010; 329:568–571 [49]
Lin^−^/Sca-1^−^/CD49f^hi^	cPten^−^/^−^	Lawson DA et al. Proc Natl Acad Sci USA 2010; 107:2610–5 [50]; Liao CP et al. Cancer Res 2010; 70:7294–7303 [51]
CD44^+^/CD133^+^/ABCG2^+^/CD24^−^	Primary tumors	Patrawala L et al. Cancer Res 2005, 65:6207–6219 [52]
PSA^−/low^/ALDH^+^/CD44^+^/α2β1^+^	Cell lines and xenografts	Salvatori L et al. PLoS One. 2012; 7(2):e31467 [53]

Finding efficient approaches to targeting prostate cancer stem-like cells is an important challenge of modern cancer research. In the recent review, Yu et al. indicate that “Each year, ~40,000 men who ‘should’ have been cured of their PCa [prostate cancer] by surgery or radiation therapy present with incurable metastatic disease that will manifest itself as metastatic lesions in the bone, usually years after primary treatment” [43]. The best currently available explanation for this is that tumor cell dissemination to the bone marrow microenvironment occurred before primary tumor removal, and disseminated tumor cells with cancer stem cell properties are responsible for a disease recurrence [43]. Consequently, eliminating prostate cancer stem-like cells may offer the potential of completely eradicating the disease [44]. Hence, defining specific cell surface markers or pathways associated with prostate cancer stem-like cells, which could potentially serve as therapeutic targets, is critical for achieving this goal [44]. However, a simple look at the currently known cell surface signatures for putative prostate cancer stem-like cells (Table 1) demonstrates that using the markers involved straightforward as potential therapeutic targets could be somewhat problematic as the same molecules are often expressed on multiple normal cell types including normal stem cells as well. Here, the fact that in prostate cancer cell surface stem cell markers such as CD44, α2β1 integrin, CD133, CD49f (α6 integrin) are all decorated with TF-Ag, which is covalently masked (either sialylated or further glycosylated) on normal cells, provides a unique opportunity to discriminate between these cell surface markers expressed on cancer stem-like cells and their normal counterparts and target prostate cancer stem cells via the TF-Ag. Further, as TF-Ag appears to be a “common denominator” for multiple prostate cancer stem-like cell populations otherwise characterized by distinct molecular signatures (Table 1), targeting prostate cancer stem-like cells via TF-Ag will endow an opportunity of attacking various species of cancer stem cells at once.

## MATERIALS AND METHODS

### Cell lines and cultures

Human prostate carcinoma cell lines PC-3 and DU-145 were from ATCC (Manassas, VA, USA). The identity of cell lines was confirmed by IDEXX BioResearch using short tandem repeat (STR) profile following the completion of the experiments. Both cell lines were routinely maintained on plastic in 5% CO_2_ humidified atmosphere using RPMI-1640 medium supplemented with L-glutamine, 10% FBS, sodium pyruvate, and non-essential amino acids.

### Isolation of cell surface TF-Ag expressing glycoproteins

For each experiment, cell surface proteins in four T75 flasks of 70–80% confluent live cultures of either PC-3 or DU-145 cells were biotinylated and purified using Pierce Cell Surface Protein Isolation Kit (Thermo Fisher Scientific Inc., Rockford, IL) according to the manufacturer instructions. Next, TF-Ag expressing glycoproteins were enriched using TF-Ag specific PNA (peanut agglutinin) lectin affinity chromatography (see Supplementary Methods for detail).

### LTQ orbitrap mass spectrometry and data analysis

For mass spectrometric analysis, isolated cell surface glycoproteins were concentrated, digested with mass spectrometry grade trypsin gold (Promega, Madison, WI) and further purified using C18 zip-tips. Tryptic peptides were loaded onto a C8 trap column (C8 CapTrap, Michrom Bioresources) and analyzed on a system with Proxeon Easy nLC system attached to the LTQ Orbitrap mass spectrometer using CID and ETD fragmentation. All MS/MS spectra were searched against the IPI database (IPI_human_20090713.fasta) using SEQUEST™ algorithm on the Sorcerer 2 integrated data appliances (IDA) server with default peak list extraction parameters. Post-search analysis was performed using the Scaffold, implementing PeptideProphet and ProteinProphet algorithms.

### Bioinformatic transmembrane topology prediction and data integration

The consensus transmembrane topology prediction strategy was adopted and three transmembrane prediction software tools including TMHMM 2.0 (http://www.cbs.dtu.dk/services/TMHMM-2.0/), MPEx 3.2 and TopPred 0.01 (http://mobyle.pasteur.fr/cgi-bin/portal.py?#forms::toppred) were used to identify the transmembrane proteins (see Supplementary Methods for detail).

### Isolation and analysis of TF-Ag positive and TF-Ag negative cells

Magnetic activated cell sorting (MACS) with biotinylated anti-TF-Ag antibody JAA-F11 [42] was used to isolate TF-Ag positive (TF+) and TF-Ag negative (TF-) prostate cancer cells (see Supplementary Methods for detail). Clonogenic survival and growth of TF+ and TF- cells was assessed as previously described [10]. Prostasphere formation of TF+ and TF- cells under non-differentiating conditions was analyzed exactly as described elsewhere [45] using 3 × 10^3^ cells per well in 12-well plates. For prostasphere formation under differentiating conditions, cells were plated and cultured as described in Supplementary Methods.

### Immunofluorescence and western blot analysis

CD44 positive prostate cancer cells were isolated by MACS using CD44 MicroBeads (Miltenyi Biotech, San Diego, CA; 130–095–194) and probed for TF-Ag expression using anti-TF-Ag antibody JAA-F11 and goat anti-mouse Alexa Fluor 594 conjugated antibody (Molecular Probes by Life Technologies, Thermo Fisher Scientific, Waltham, MA; Cat # A11020). For Western blot analysis, see complete list of antibodies used in Supplementary Methods.

## SUPPLEMENTARY MATERIALS TABLE


