# Supplementary material for: Cell surface Thomsen-Friedenreich proteome profiling of metastatic prostate cancer cells reveals potential link with cancer stem cell-like phenotype
Source: Oncotarget. 2017 Oct 20;8(58):98598–608. doi: 10.18632/oncotarget.21985 (PMC5716753; doi:10.18632/oncotarget.21985)
Supplement: Supplementary file 1 [file oncotarget-08-98598-s001.pdf]

Cell surface Thomsen-Friedenreich proteome profiling of metastatic prostate cancer cells reveals potential link with cancer stem cell-like phenotype

SUPPLEMENTARY MATERIALS

Isolation of cell surface TF-Ag expressing glycoproteins, LTQ orbitrap mass spectrometry and data analysis

For each experiment, cell surface proteins in four T75 flasks of 70–80% confluent live cultures of either PC-3 or DU-145 cells were biotinylated and purified using Pierce Cell Surface Protein Isolation Kit (Thermo Fisher Scientific Inc., Rockford, IL) according to the manufacturer instructions. Next, TF-Ag expressing glycoproteins were enriched using TF-Ag specific PNA (peanut agglutinin) lectin affinity chromatography as follows. A 2 ml Pierce centrifuge column was packed with 1 ml of agarose-bound PNA (Vector Laboratories Inc., Burlingame, CA). The column was washed twice with 3 ml of binding buffer (10 mM HEPES, pH 7.5, 150 mM NaCl, 1 mM MgCl2, 1 mM CaCl2, and 1 mM MnCl2). Biotinylated proteins released by DTT were incubated with agarose-bound PNA lectin column for 1 hour on a rotator at room temperature. The column was washed twice with 3 ml of binding buffer to remove nonspecifically bound proteins. Finally, captured biotinylated glycoproteins were released with 3 mL of elution buffer (200 mM D-galactose in binding buffer, pH 7.5). This step was repeated twice, and the eluted fractions were pooled. Pooled fractions were concentrated with Microcon YM-10 centrifugal filter devices, reduced with DTT (2 mM final concentration) for 30 minutes, and alkylated with iodoacetamide (25 mM final concentration) for 30 min on ice in the dark. Isolated peptides were digested with mass spectrometry grade trypsin gold (Promega, Madison, WI) at 37 °C overnight. The digested reaction was stopped with 50 µl of 10 % TFA, the liquid phase was added (200 µl acetonitrile), and evaporated using a SpeedVac.

Dried sample was resuspended in 15 µl 1% (v/v) formic acid in Milli-Q water. Before mass analysis, the peptides fragments were further purified using C18 zip-tips to remove excess salt. The peptides were eluted from the zip-tip in 5 µl of 70% acetonitrile, 1% formic acid, lyophilized, and resuspended in 8 µl 1% formic acid. Resuspended solution was centrifuged at max speed for 10 minutes and 7.5 µl removed to an autosampler vial. The vials were placed in a thermostatted (4°C) autosampler. An aliquot (5 µl) of each sample was loaded onto a C8 trap column (C8 CapTrap, Michrom Bioresources). Bound peptides were eluted from the trap column onto a 10.5cm, 150µm i.d. pulled-needle analytical column packed with magic C18 reversed phase resin (Michrom Bioresources). Peptides were separated and eluted from the analytical column with a continuous gradient of acetonitrile from 5 to 45% (in 0.1% formic acid) over 80 minutes. The Proxeon Easy nLC system is attached to an LTQ Orbitrap mass spectrometer. Following a high-resolution FTMS scan of the eluting peptides, each second, the 9 most abundant peptides were subjected to peptide fragmentation (CID in iontrap and ETD in the C-trap). Data across a total of 100 minutes of elution were collected and then searched against the IPI Human database.

All MS/MS spectra were searched against the IPI database (IPI_human_20090713.fasta). The search was performed using SEQUEST™ algorithm on the Sorcerer 2 integrated data appliances (IDA) server with default peak list extraction parameters, and post-search analysis was performed using Scaffold implementing PeptideProphet and ProteinProphet algorithms. The search parameters were as follows: semienzyme digest using trypsin (after Lys or Arg) with up to two missed cleavages; monoisotopic precursor mass range of 400–5000 amu; fixed modification, Carbamidomethyl of C; variable modification, oxidation of M and 3-sulfanylpropanoyl modification on K and N-term (UniMod #126); peptide ion mass tolerance 25 ppm; fragment ion mass tolerance 1 amu; peptide charges +1, +2, and +3; fragment mass type was set to monoisotopic, and the maximum number of modifications was set to four per peptide. Advanced search options that were enabled included the following: XCorr score cutoff of 1.5, isotope check using a mass shift of 1.003355 amu, decoy peptide generation with 2 × mass tolerance expansion for decoy masses. The database search results were first validated using the PeptideProphet software, and then the peptides were assigned for protein identification using the ProteinProphet software. In this study, the criteria for identification was set to 2 peptide minimum, 95% ID confidence on peptide, mass error on peptide < 10 ppm, 99% identification confidence on protein. This resulted in an overall false positive rate below 1%. Identifications were examined using the Scaffold 3.5.1.

Bioinformatic transmembrane topology prediction and data integration

Lists of identified proteins from each experiment were exported from Scaffold 3.5.1 to an Excel format file through exporting Protein Report function. The IPI accession numbers were used to search the EBI IPI database with the EBI Dbfetch function. The returned results were formatted as raw style and fasta format and saved as a fasta format file. Each fasta format file served as the data source for analysis of transmembrane segments.

The consensus transmembrane topology prediction strategy was adopted and three transmembrane prediction software tools were used to identify transmembrane proteins including TMHMM 2.0 (http://www.cbs.dtu.dk/services/TMHMM-2.0/), MPEx 3.2, and TopPred 0.01 (http://mobyle.pasteur.fr/cgi-bin/portal.py?#forms::toppred). The fasta format files (multiple protein sequences) were uploaded to the TMHMM and TopPred server and analyzed using the default settings. The lists of predicted transmembrane proteins were copied and saved into separate files. For MPEx analysis pipeline, each multiple protein sequence fasta format file was split into multiple fasta format files (with a single IPI fasta format record in one file) under the same directory by mEMBOSS program seqretsplit and then analyzed by MPEx. The threshold for positive predicted transmembrane protein was one transmembrane segment predicted by TMHMM, two transmembrane segments by MPEx, and three transmembrane segments by TopPred. A predicted transmembrane protein was counted as a “true” transmembrane protein when it was predicted by TMHMM or by both MPEx and TopPred.

The experiment results from PC-3 and DU-145 cell lines were combined together. Every “true’ transmembrane protein from the same experiment was saved in one file and imported to Access database. Every “true” transmembrane protein was designated as TF-antigen expressing glycoprotein if it appeared in at least two experimental datasets. Otherwise, the transmembrane protein was designated as potential TF-antigen carrier.

Isolation and analysis of TF-Ag positive and TF-Ag negative cells

Magnetic activated cell sorting (MACS) with biotinylated anti-TF-Ag antibody JAA-F11 (42) was used to isolate TF-Ag positive (TF+) and TF-Ag negative (TF-) prostate cancer cells. Purified mouse monoclonal JAA-F11 were biotinylated using ImmunoProbe Biotinylation Kit BK101 (Sigma-Aldrich, St. Louis, MO) according to the manufacturer instruction. The ability of the biotinylated antibody to bind TF-Ag in a dose dependent manner was confirmed using ELISA against immobilized TF-Ag. To purify TF+ and TF- cells 1 × 107 prostate cancer cells (PC-3 or DU-145) grown in DMEM High Glucose Medium and 20 µl of 0.5 mg/ml biotinylated JAA-F11 antibody (1 µg of antibody per 1 × 106 cells) were used in conjunction with Anti-Biotin MicroBeads (Miltenyi Biotec, San Diego, CA; Catalog number 130–090–485) according to the manufacturer instructions for each round of cell isolation.

Clonogenic survival and growth of TF+ and TF- cells was assessed as previously described (10). Briefly, TF+ and TF- cells were plated at low density (200 viable cells per well) in quadruplicate in a 24-well culture plate in complete media. Seven days later, the cells were fixed with 1% formaldehyde in PBS, stained with hematoxylin, and colonies of more than 15 cells were scored.

Prostasphere formation of TF+ and TF- cells under non-differentiating conditions was analyzed exactly as described elsewhere (45) using 3 × 103 cells per well in 12-well plates. Briefly, freshly isolated TF+ and TF- cells were suspended in Prostate Epithelial Cell Growth Medium (PrEGM, Lonza, Walkersville, MD; Cat # CC-3166) at the concentration of 3 × 104 cell/ml. Next, 160µl of cell suspension was mixed with 240µl of Matrigel (BD Biosciences, Cat # 354234) and 200 µl of the mixture per well were plated around the rim in a 12-well plate. The plates were placed in a 37 °C incubator for 30 min to allow Matrigel to solidify and 800 µl of PrEGM was added to each well. The plates were incubated for two weeks in a 37 °C humidified incubator with 5% CO2 atmosphere with half of the media being exchanged every 3 days and cells being observed and photographed on the inverted microscope.

For prostasphere formation under differentiating conditions, 600 cell per well of TF+ and TF- cells were plated in 6-well ultralow attachment plate in triplicate using complete DMEM High Glucose Medium supplemented with 10% FBS. The cells were incubated for two weeks in a 37 °C humidified incubator with 5% CO2 atmosphere with half of the media being exchanged every 3 days and cells being observed and photographed on the inverted microscope.

Immunofluorescence and western blot analysis

For immunofluorescence analysis, CD44 positive prostate cancer cells were isolated by MACS using CD44 MicroBeads (Miltenyi Biotech, San Diego, CA; Cat # 130–095–194) according to the manufacturer instructions and probed for TF-Ag expression using anti-TF-Ag antibody JAA-F11 and goat anti-mouse Alexa Fluor 594 conjugated antibody (Molecular Probes by Life Technologies, Thermo Fisher Scientific, Waltham, MA; Cat # A11020).

For immunoprecipitation and Western blot confirmation of TF-Ag expression, individual glycoproteins were precipitated from 3 mg of total protein lysates of PC-3 or DU-145 cells using 10 µg of the following antibodies and Dynabeads Protein G

(Invitrogen, Thermo Fisher Scientific, Waltham, MA; Cat # 10003D): anti-CD44 clone 156–3C11 (Cell Signaling Technology, Boston, MA); anti-Integrin β1 sc-59829, anti-Integrin α2 sc-53506, anti-Integrin α6 sc-53365, anti-Syndecan-1 sc-7099, anti-CD98 sc-7095, anti-CD59 sc-59095 (all Santa Cruz Biotechnology, Santa Cruz, CA), anti-Transferrin Receptor ab109259 (Abcam Ltd., Cambridge, MA); anti-CD133 MBS462020 (MyBioSource, San Diego, CA). The targeted protein complexes were eluted from Protein G Agarose with 50 mm glycine-HCl pH 2.7 and resolved on a NuPAGE mini gel (Invitrogen, Thermo Fisher Scientific, Waltham, MA), transferred to a nitrocellulose membrane (Invitrogen), and probed with mouse anti-TF-Ag monoclonal antibody JAA-F11 at 1:100 dilution followed by HRP-conjugated anti-mouse secondary antibody and enhanced chemiluminescent detection. After that, the membranes were stripped with β-ME stripping buffer (62.5 mM Tris-Cl pH 6.8, 2% SDS, 100 mM β-mercaptoethanol) at 50 °C for 30 minutes. Following stripping, the membranes were re-probed with the primary antibodies that were used for immunoprecipitation to confirm the proper position of TF-AG reactive bands. In Western blot analysis the primary antibodies from Santa Cruz were used at 1:200 dilution, and all other at 1:1000 dilution.

Supplementary Table 1: Potential TF-antigen expressing transmembrane or secreted proteins and TF-antigen interacting proteins

Accession Number	Protein Name	Location
IPI00219219.3	Galectin-1	Secreted
IPI00465431.8	Galectin-3	Secreted
IPI00936444.2	Mucin-5B	Secreted
IPI00103397.2	Mucin-5 AC	Secreted
IPI00385172.5	MUCIN-1 ISOFORM 13 PRECURSOR	Secreted
IPI00215995.1	ISOFORM 1 OF INTEGRIN ALPHA-3	Transmembrane
IPI00306604.5	INTEGRIN ALPHA-5	Transmembrane
IPI00027422.2	ISOFORM BETA-4C OF INTEGRIN BETA-4	Transmembrane
IPI00028911.2	DYSTROGLYCAN	Transmembrane
IPI00031713.2	CD70 ANTIGEN	Transmembrane
IPI00008494.4	INTERCELLULAR ADHESION MOLECULE 1	Transmembrane
IPI00152850.2	JUNCTIONAL ADHESION MOLECULE 3 PRECURSOR	Transmembrane
IPI00152540.3	ISOFORM 1 OF CD109 ANTIGEN	Transmembrane
